# First person – Setsuya Minami

**DOI:** 10.1242/bio.050443

**Published:** 2020-01-24

## Abstract

First Person is a series of interviews with the first authors of a selection of papers published in Biology Open, helping early-career researchers promote themselves alongside their papers. Setsuya Minami is first author on ‘BAG6 contributes to glucose uptake by supporting the cell surface translocation of the glucose transporter GLUT4’, published in BIO. Setsuya is a PhD student in the lab of Hiroyuki Kawahara at Tokyo Metropolitan University, Japan, investigating how proteins cause diseases.


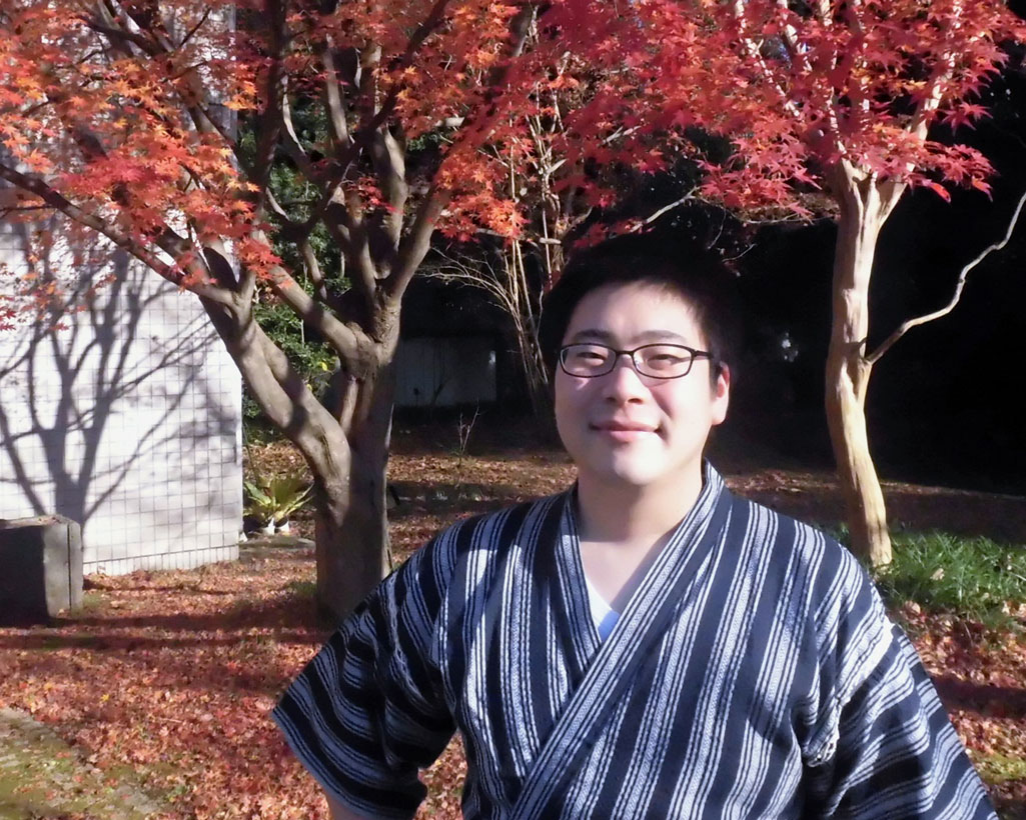


**Setsuya Minami**

**What is your scientific background and the general focus of your lab?**

I am a pharmacist, however, I am very interested in molecular cell biology. A new finding in this area makes the discovery of new treatment methods and drugs for diseases possible. Therefore, I decided to join our lab, which focuses on the function of protein. In the lab, I tried to examine the molecular mechanism of GLUT4 protein translocation, which is deeply related to diabetes.

“A new finding in [molecular cell biology] makes the discovery of new treatment methods and drugs for diseases possible.”

**How would you explain the main findings of your paper to non-scientific family and friends?**

Diabetes is a condition in which high blood glucose is exhibited and is a major problem in many developed countries. Our bodies possess mechanisms to keep blood sugar levels constant. One of them is insulin-responsive glucose uptake in the cells. When insulin is released into the blood, GLUT4, a glucose transporter that is usually stored inside the cells, is translocated to the cell surface, and this stimulates glucose incorporation into these cells. As a result, blood sugar is maintained at a normal level. It is known that defects in GLUT4 trafficking can develop into type 2 diabetes. I found new molecular machinery that supports accurate GLUT4 trafficking inside the cells.

**What are the potential implications of these results for your field of research?**

From the viewpoint of medical progress, my finding may trigger the proposal of a new therapeutic target of type 2 diabetes. In the future, I believe that BAG6-mediated GLUT4 control will save a lot of patients with diabetes. From the viewpoint of basic science, our laboratory previously proposed that BAG6 controls intracellular trafficking of endosomal proteins via Rab family protein degradation. In this study, we propose that BAG6 supports GLUT4 trafficking. These results collectively suggest the BAG6 might contribute to the intracellular trafficking of GLUT4 by controlling Rab8a. Since defects in intracellular trafficking are critical for a wide variety of diseases, BAG6-mediated vesicle trafficking control might become an interesting research area in the future.

“[…] my repeated experiments gradually changed the hypothesis to experimental facts.”

**What has surprised you the most while conducting your research?**

My start point of this study was inspired by the reports describing polymorphisms of the BAG6 gene present in diabetic patients. Although there was little experimental evidence that BAG6 could be involved in GLUT4 trafficking, my repeated experiments gradually changed the hypothesis to experimental facts. I was impressed to see that the function of BAG6 indeed expanded to an unexpected area in GLUT4 trafficking. It was surprising for me to go from picking up small pieces of evidence to this novel finding.

**What, in your opinion, are some of the greatest achievements in your field and how has this influenced your research?**

At the initial stage of this work, it was difficult for me to evaluate the insulin-stimulated GLUT4 translocation, and I needed a lot of time to establish a reliable experimental system for this study. Studying previously reported great observations and the quantification system of GLUT4 was really helpful. A process of scrutinizing and brushing up my research system was a good opportunity for me. At the time of submission of this paper, I could be confident with my results in myself.

**Figure:**
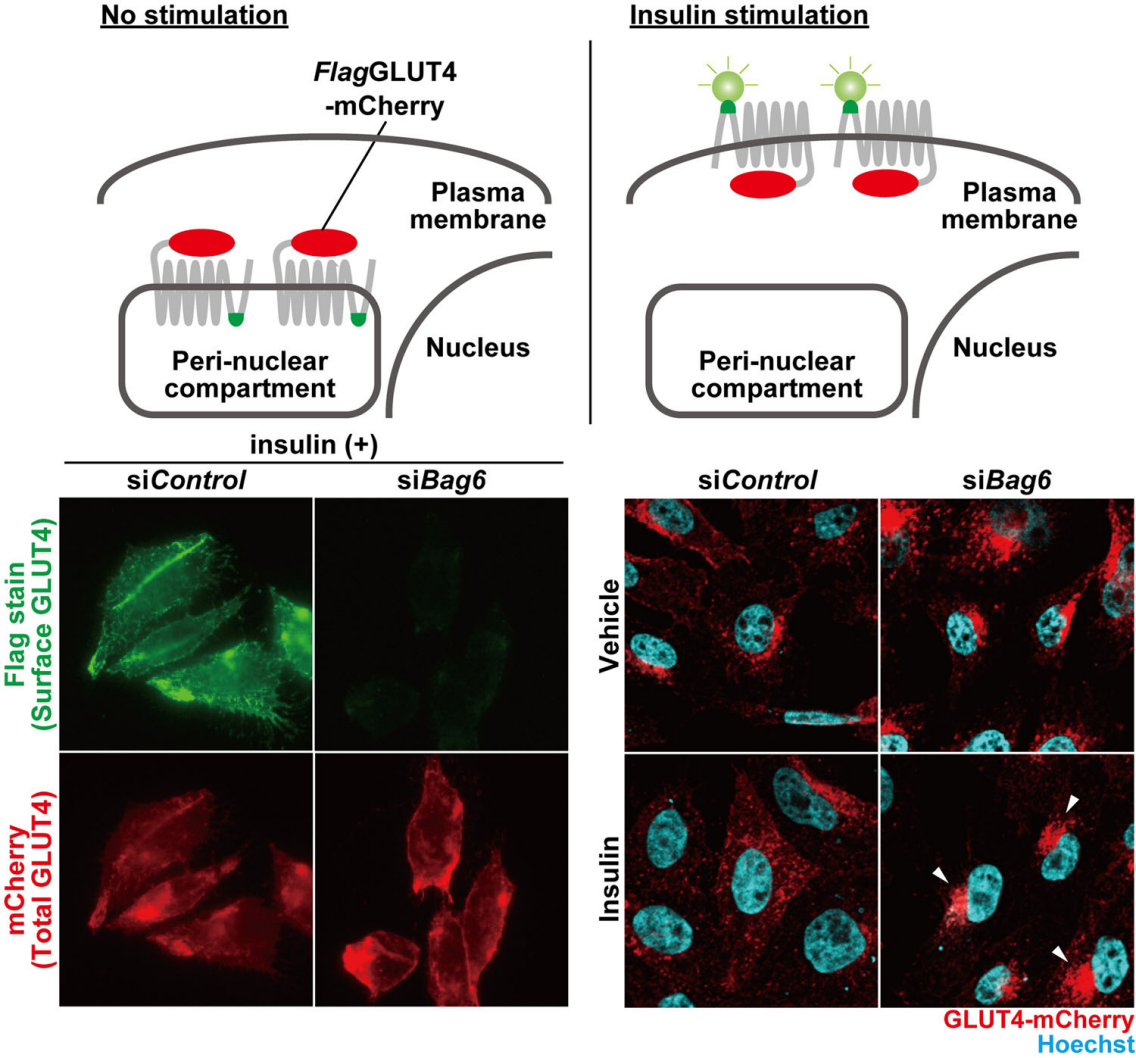
**Cell surface GLUT4 detection system (upper panel) and GLUT4 translocation with insulin-stimulation in BAG6-suppressed cells (downer panel).**

**What changes do you think could improve the professional lives of early-career scientists?**

I respect pioneers in Japanese scientific research. They often talk about how the government should provide various kinds of support to early-career researchers. I think too that we need several kids of support. For example, a scholarship system with low burden, stable employment with little anxiety, enough salary to live on and research funds for young researchers. These anxiety factors may have hindered the development of diverse studies. I wish for Japanese scientific research to be as active as the rest of the world's and from now on. To this end, it is important to satisfy the environment surrounding the research of young researchers.

**What's next for you?**

I am going to focus on education in the next generation. I believe that my experiences have a positive impact on the youth and can bring them a bright future.
